# The Role of Spermidine Synthase (SpdS) and Spermine Synthase (Sms) in Regulating Triglyceride Storage in *Drosophila*

**DOI:** 10.3390/medsci9020027

**Published:** 2021-05-02

**Authors:** Tahj S. Morales, Erik C. Avis, Elise K. Paskowski, Hamza Shabar, Shannon L. Nowotarski, Justin R. DiAngelo

**Affiliations:** Division of Science, Pennsylvania State University, Berks Campus, Reading, PA 19610, USA; tsm5324@psu.edu (T.S.M.); erik.c.avis@gmail.com (E.C.A.); ekp5150@outlook.com (E.K.P.); hqs5372@psu.edu (H.S.)

**Keywords:** spermidine synthase, spermine synthase, Drosophila, triglyceride, fat body

## Abstract

Polyamines are small organic cations that are important for several biological processes such as cell proliferation, cell cycle progression, and apoptosis. The dysregulation of intracellular polyamines is often associated with diseases such as cancer, diabetes, and developmental disorders. Although polyamine metabolism has been well studied, the effects of key enzymes in the polyamine pathway on lipid metabolism are not well understood. Here, we determined metabolic effects resulting from the absence of spermidine synthase (*SpdS*) and spermine synthase (*Sms*) in *Drosophila*. While *SpdS* mutants developed normally and accumulated triglycerides, *Sms* mutants had reduced viability and stored less triglyceride than the controls. Interestingly, when decreasing *SpdS* and *Sms*, specifically in the fat body, triglyceride storage increased. While there was no difference in triglycerides stored in heads, thoraxes and abdomen fat bodies, abdomen fat body DNA content increased, and protein/DNA decreased in both *SpdS-* and *Sms-RNAi* flies, suggesting that fat body-specific knockdown of *SpdS* and *Sms* causes the production of smaller fat body cells and triglycerides to accumulate in non-fat body tissues of the abdomen. Together, these data provide support for the role that polyamines play in the regulation of metabolism and can help enhance our understanding of polyamine function in metabolic diseases.

## 1. Introduction

Metabolism-related diseases have been increasing over the past three decades, with obesity becoming one of the most prevalent diseases; approximately one-third of the world’s population is considered obese [1]. Obesity is characterized by increased fat accumulation and is associated with a number of comorbidities such as heart disease and type II diabetes [2]. Due to the substantial presence of obesity globally, understanding all of the genes and biological pathways which are involved in regulating the storage of fat in humans is important to allow us to develop treatments and therapeutics for this disease.

Polyamines are small, organic cations that have been shown to be important for regulating cell growth, proliferation, cell cycle progression, and apoptosis [3,4]. Polyamine synthesis begins with the conversion of ornithine to putrescine by ornithine decarboxylase (Odc1). Putrescine is then converted into spermidine by the enzyme spermidine synthase (SpdS) and finally, spermidine is converted into spermine by spermine synthase (Sms) [4]. While the functions of polyamines in regulating disease states such as cancer have been studied in detail [5], less is known about how polyamines function in regulating lipid metabolic processes. Polyamines have been shown to be essential for adipocyte differentiation, because treating 3T3-L1 preadipocytes in culture with the Odc1 inhibitor α-difluoromethylornithine (DFMO) or the spermine synthase inhibitor N-(3-aminopropyl)-cyclohexylamine (APCHA) inhibits differentiation of these cells into mature adipocytes [6,7,8]. Moreover, treating mature adipocytes in culture with APCHA decreases triglyceride levels [8]. Interestingly, in mice fed a high-fat diet, injection of either spermidine or spermine reduced weight gain [9,10], suggesting that polyamines can function to limit fat accumulation in an intact organism.

The fruit fly, *Drosophila melanogaster*, has recently emerged as a model organism to study human obesity and other metabolic diseases due to its similar genetics and physiology to humans [11,12]. Flies also make polyamines and the enzymes involved in their synthesis are conserved with humans [13]. Our lab has previously shown that flies heterozygous for the *Odc1* gene accumulate triglycerides, which is due to a combination of increasing numbers of fat cells as well as the amount of triglyceride stored in fat body cells [14]. In addition, decreasing *Odc1* results in increased expression of the genes coding for the major lipid synthesis enzymes, *fatty acid synthase* (*FASN*) and *acetyl-CoA carboxylase* (*ACC*) [14]. While the metabolic functions of fly *Odc1* have been described, whether *SpdS* or *Sms* function in *Drosophila* to regulate lipid storage is unknown.

In this study, we characterized the roles of the polyamine synthesis enzymes SpdS and Sms in regulating lipid storage in *Drosophila*. Decreasing *SpdS* throughout the entire organism, or specifically in the fat body, resulted in the accumulation of triglycerides. However, while *Sms* mutants had blunted fat storage, fat body-specific decreases in *Sms* led to lipid accumulation. Interestingly, the lipid accumulation phenotypes that resulted from fat body-specific decreases in either *SpdS* or *Sms* resulted from an increase in triglycerides in non-fat body cells or tissues of the abdomen, because triglyceride storage per abdomen fat body and combined head–thorax was unchanged in *SpdS-RNAi* and *Sms-RNAi* flies. However, decreasing *SpdS* and *Sms* in the fat body led to an increase in the number of fat cells present in the abdomen fat body that were smaller in size than control fat body cells. Together, these data suggest a role for both *SpdS* and *Sms* in regulating the storage of triglycerides and strengthen the link between polyamines and lipid homeostasis. 

## 2. Materials and Methods

### 2.1. Fly Genetics

The fly lines utilized in these experiments and their Bloomington Stock Center ID (listed in parentheses) are as follows: SpdS -/-: y[1] w[*]; Mi{y[+Dint2]=MIC}SpdS[MI00898]/TM3, Sb[1] Ser[1] (BL#34119), SpdS -/- control: *y[1]w[1]* (BL#1495), Sms -/-: w[*]; P{GSV1}Sms[C909] (BL#43396), Sms -/- control: *w^1118^* (BL#3605), UAS-Sms-RNAi: y[1] sc[*] v[1] sev[21]; P{TRiP.HMC03665}attP40 (BL#52924), UAS-SpdS-RNAi: y[1]sc[*]v[1] sev[21]; P{TRiP.HMC04307}attP40 (BL#56011), UAS-EGFP-RNAi: y[1]sc[*]v[1]sev[21]; P{y[+t7.7]v[+t1.8]=VALIUM20-EGFP.shRNA.1}attP40 (BL#41555), *yolk*-*Gal4* [15]. The *SpdS* and *Sms* mutant alleles used here are considered hypomorphic as *SpdS* and *Sms* expression, respectively, is decreased, but not completely ablated (data not shown). Flies were raised in a 12 h:12 h light:dark cycle at 25 °C on a standard cornmeal–sucrose medium (100 mL Karo Lite Corn Syrup (ACH Food Companies, Inc., Oakbrook Terrace, IL, USA), 65 g cornmeal, 40 g sucrose, 9 g *Drosophila* agar (Genesee Scientific, San Diego, CA, USA), and 25 g whole yeast in 1.25 L water).

### 2.2. Protein, Triglyceride and DNA Assays

One-week-old whole female flies or dissected cuticles from abdomens with fat bodies attached from one-week-old female flies, or combined heads and thoraxes with legs and wings attached from one-week-old female flies, were homogenized in lysis buffer (140 mM NaCl, 50 mM Tris-HCl, pH 7.5, 0.1% Triton-X with 1X protease inhibitor cocktail (Millipore-Sigma, Burlington, MA, USA)). Following homogenization, samples were centrifuged at 4 °C for 15 min at 16,000× *g*. Proteins, triglycerides, and DNA were measured as previously described [16]. Proteins were measured using the Pierce BCA Protein Assay kit (ThermoFisher, Waltham, MA, USA), and triglycerides were measured using the Infinity Triglyceride kit (ThermoFisher) in all samples. Triglyceride measurements were normalized to total protein content. In fat body samples, DNA content was measured using the Quant-iT double-stranded DNA high-sensitivity kit, according to the manufacturer’s instructions (ThermoFisher). 

### 2.3. Statistics

The results are expressed as the mean ± standard error (SE). Comparisons between experimental and control conditions were made using unpaired Student’s *t*-tests or one-way analysis of variance (ANOVA), as described in the figure legends. *p* < 0.05 was considered statistically significant.

## 3. Results

### 3.1. SpdS and Sms Mutants Have Altered Triglyceride Storage

**Figure 1:**
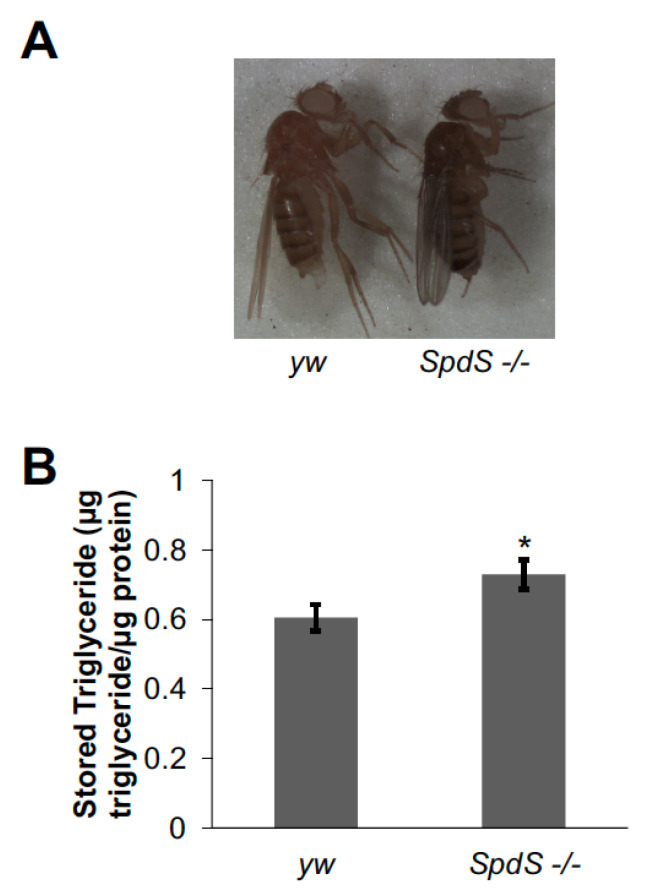
*Spermidine synthase* (*SpdS*) mutant flies have augmented triglyceride storage. (**A**) One-week-old female *SpdS -/-* flies had a larger abdomen compared to *yw* controls. A representative image is shown. (**B**) Triglycerides were measured in *SpdS -/-* mutants by homogenizing pairs of one-week-old adult female flies and *yw* controls. Triglyceride measurements were normalized by dividing by total protein content. Bars represent means ± S.E. (*n* = 23–25). * *p* < 0.05 using Student’s *t*-test comparing *SpdS -/-* to *yw* controls.

In order to study the effects of *SpdS* and *Sms* on fly lipid metabolism, we characterized mutants of *SpdS* (*SpdS^MI00898^*, referred to here as *SpdS -/-*) and *Sms* (*Sms^c909^*, referred to here as *Sms -/-*) and compared them to *yw* and *w^1118^* flies, respectively. *SpdS -/-* flies had similar viability and developmental progression compared to controls (data not shown). However, *SpdS -/-* adults appeared larger by eye, especially in the abdomen (Figure 1A). It is possible that any increase in size in *SpdS -/-* flies was due to increased lipid storage. To test this hypothesis, triglyceride content was measured in *SpdS -/-* flies and compared to controls. Triglyceride content was noticeably increased in *SpdS -/-* flies (Figure 1B), indicating a role for *SpdS* in limiting fat accumulation in flies.

**Figure 2:**
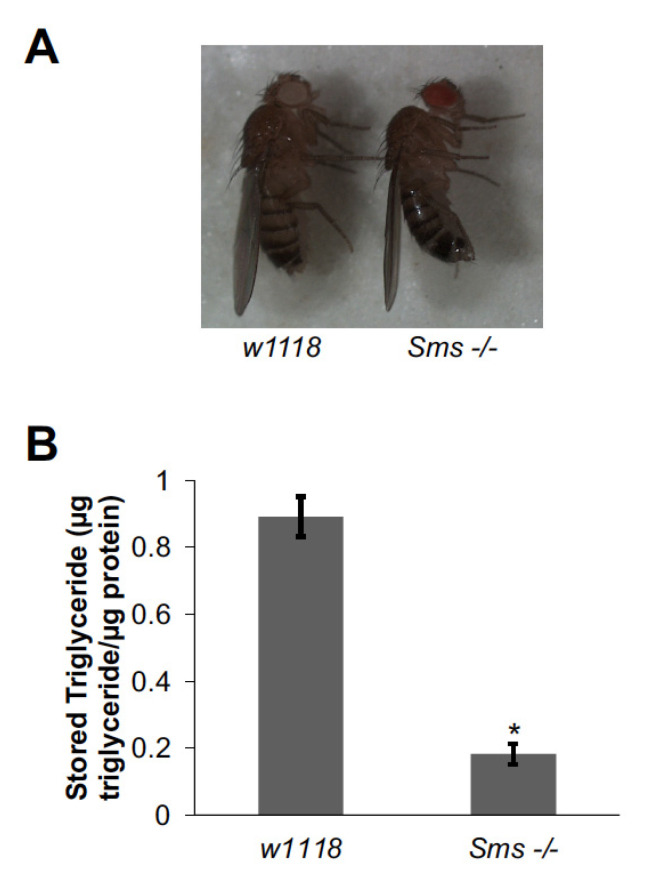
*Spermine synthase* (*Sms*) mutant flies store less triglyceride than control flies. (**A**) One-week-old female *Sms -/-* flies displayed a smaller abdomen compared to *w1118* controls. A representative image is shown. (**B**) Triglycerides were measured in *Sms -/-* mutants by homogenizing pairs of one-week-old adult female flies and *w1118* controls. Triglyceride measurements were normalized by dividing by total protein content. Bars represent means ± S.E. (*n* = 22–25). * *p* < 0.05 using Student’s *t*-test comparing *Sms -/-* to *w1118* controls.

In contrast to *SpdS -/-* flies, but consistent with previous reports, *Sms -/-* flies had a 47% reduction in viability (data not shown [17]). However, the adult *Sms -/-* flies that did emerge looked smaller by eye and had thinner abdomens (Figure 2A). To determine whether the thinner abdomen was due to decreased fat storage, triglyceride content was measured in *Sms -/-* flies. *Sms -/-* flies had lower triglyceride levels compared to controls (Figure 2B), a phenotype opposite of the *SpdS -/-* flies (Figure 1B). Together, these data combined with the *SpdS -/-* data described above suggest a role for both *SpdS* and *Sms* in regulating lipid metabolism in *Drosophila*.

### 3.2. SpdS and Sms Act in the Fat Body to Regulate Organismal Lipid Storage

**Figure 3:**
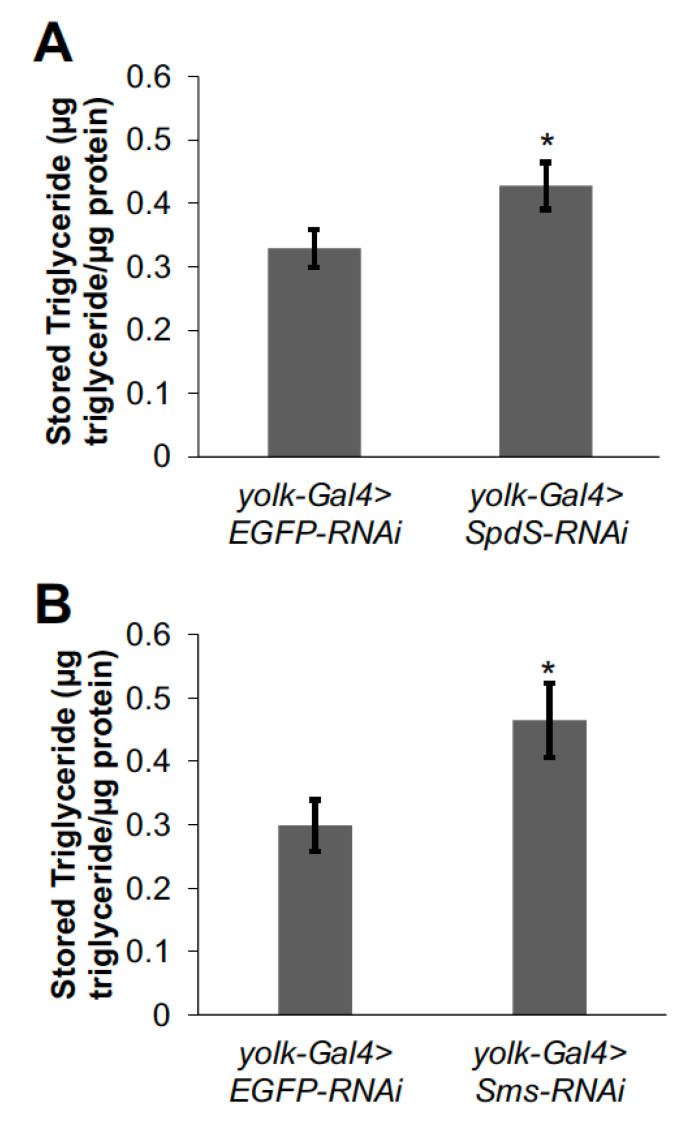
Decreasing either *SpdS* or *Sms* in the fat body increases triglyceride storage. Triglycerides were measured in (**A**) one-week-old female *yolk-Gal4>SpdS-RNAi* flies and *yolk-Gal4>EGFP-RNAi* controls (*n* = 49–60) and (**B**) one-week-old female *yolk-Gal4>Sms-RNAi* flies and *yolk-Gal4>EGFP-RNAi* controls (*n* = 19–20). Triglyceride measurements were normalized by dividing by total protein content. Bars represent means ± S.E. * *p* < 0.05 using Student’s *t*-test comparing *yolk-Gal4>SpdS-RNAi* or *yolk-Gal4>Sms-RNAi* flies to the appropriate *yolk-Gal4>EGFP-RNAi* controls.

The *Sms -/-* flies had reduced viability and other developmental defects [17]; therefore, it is possible that the lower lipid levels in these flies may be secondary to one of these developmental defects. To rule out this possibility, as well as to assess whether *SpdS* and *Sms* act in the fat body (the major lipid storage organ in the fly) to regulate lipid metabolism, we used RNA interference (RNAi) to decrease *SpdS* or *Sms* specifically in the adult fat body. RNAi knockdown of *SpdS* in the fly fat body resulted in increased triglyceride levels (Figure 3A), consistent with the lipid storage phenotype of the *SpdS -/-* flies (Figure 1B). In contrast, the fat body-specific RNAi knockdown of *Sms* revealed increased triglyceride content (Figure 3B), which is the opposite of the *Sms -/-* phenotype and suggests that the blunted lipid storage phenotype in the *Sms -/-* flies may be indirect or secondary to a developmental defect. Overall, these data suggest that both *SpdS* and *Sms* normally act in the fly fat body to regulate the storage of triglycerides.

### 3.3. SpdS and Sms Control Fat Body Cell Number and Size

**Figure 4:**
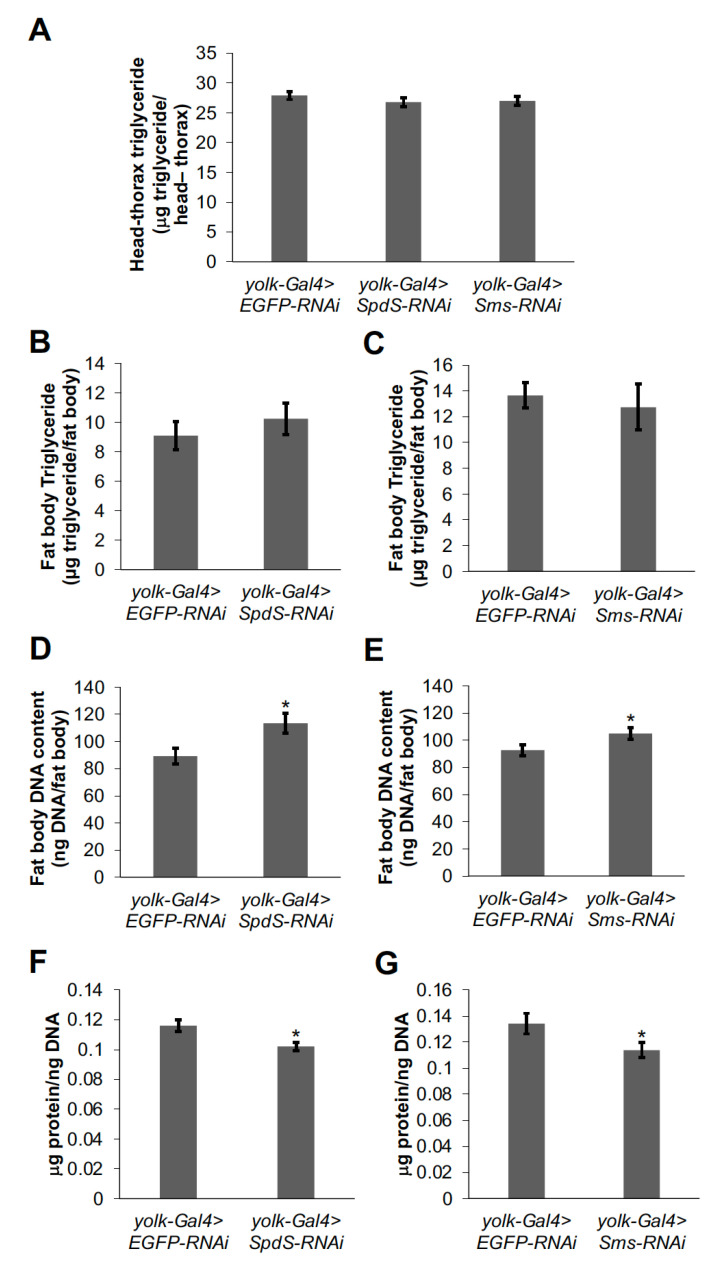
Decreasing either *SpdS* or *Sms* in the fat body increases the number but decreases the size of fat body cells. (**A**) Total triglyceride was measured in samples of combined heads and thoraxes dissected from one-week-old female *yolk-Gal4>SpdS-RNAi* and *yolk-Gal4>Sms-RNAi* flies and *yolk-Gal4>EGFP-RNAi* controls (*n* = 24–48). A one-way ANOVA was used to compare head–thorax triglycerides in *yolk-Gal4>SpdS-RNAi* and *yolk-Gal4>Sms-RNAi* flies, and *yolk-Gal4>EGFP-RNAi* controls. (**B**,**C**) Total triglyceride, (**D**,**E**) total DNA, and (**F**,**G**) total protein contents were measured in fat bodies dissected from one-week-old female *yolk-Gal4>SpdS-RNAi* flies and *yolk-Gal4>EGFP-RNAi* controls (*n* = 21–26) and fat bodies dissected from one-week-old female *yolk-Gal4>Sms-RNAi* flies and *yolk-Gal4>EGFP-RNAi* controls (*n* = 54–55). Protein measurements were normalized by dividing by total DNA content to determine the size of each fat body cell. Bars represent means ± S.E. * *p* < 0.05 using Student’s *t*-test comparing *yolk-Gal4>SpdS-RNAi* or *yolk-Gal4>Sms-RNAi* flies to the appropriate *yolk-Gal4>EGFP-RNAi* controls.

To better understand the mechanism of how *SpdS* and *Sms* act in the fly fat body to regulate lipid metabolism, we dissected combined heads and thoraxes as well as abdomen fat bodies from *SpdS-RNAi* and *Sms-RNAi* flies and measured the amount of triglyceride stored in each combined head–thorax and abdomen fat body preparation. Interestingly, there were no differences in triglyceride levels in *SpdS*-*RNAi* or *Sms*-*RNAi* fat bodies or head–thorax preparations and their respective controls (Figure 4A–C). This suggests that the increased triglyceride storage phenotype of whole *SpdS-RNAi* and *Sms-RNAi* flies arises from excess lipids being stored in non-fat body cells/tissues in the abdomen. Moreover, we have previously shown that fat body DNA content can be used as a surrogate measurement for fat body cell number, and protein/DNA can be used as an indication of cell size [16,18]; therefore, we measured fat body DNA content and protein in fat bodies with decreased *SpdS* or *Sms* levels. Interestingly, in both *SpdS-RNAi* and *Sms-RNAi* fat bodies, fat body DNA content increased compared to control flies (Figure 4D,E), but protein/DNA decreased compared to controls (Figure 4F,G). Together, these data suggest a potential role of *SpdS* and *Sms* in regulating the number and size of abdomen fat body cells.

## 4. Discussion

In this study, we uncovered a role for SpdS and Sms enzymes in regulating lipid metabolism in the fruit fly, *Drosophila melanogaster*. While *SpdS -/-* flies accumulated triglycerides, *Sms -/-* flies were lean compared to controls. Moreover, previous studies using an independent *Sms* mutant in *Drosophila* have shown that the loss of *Sms* reduces viability, and our data are consistent with this model because we observed a 47% reduction in viability in our *Sms* mutants (data not shown [17]). Loss of *Sms* also results in neuronal deterioration due to impaired autophagy flux and mitochondrial function [17]. Therefore, it is possible that the lower triglyceride storage phenotype that we observed in *Sms -/-* flies here was secondary to a defect that emerges during development (a defect that may be dependent on the levels of polyamines in these animals). Consistent with this hypothesis, when *Sms* expression is decreased, specifically in the adult fat body, we showed that triglycerides accumulate, which is similar to the phenotypes observed in both *SpdS -/-* and *SpdS-RNAi* flies. This suggests that *SpdS* and *Sms* both act in the *Drosophila* fat body to regulate the storage of triglycerides.

Previous studies from our lab have shown that while *Odc1* mutants in *Drosophila* are not viable, *Odc1* heterozygous flies have excess triglyceride storage, and part of this phenotype arises from increased fat cell number [14]. This increase in fat cell number is consistent with the *SpdS-RNAi* and *Sms-RNAi* fat body phenotypes described here, suggesting that these three polyamine synthesis enzymes may work in concert to regulate lipid metabolism in the fly fat body (but whether the production of the polyamines themselves contributes to regulating lipid metabolism in the fat body is unclear). However, *Odc1* heterozygous flies have a stronger lipid accumulation phenotype than *SpdS-RNAi* and *Sms-RNAi* flies, much of which comes from increased triglyceride storage in each fat cell [14], indicating that *Odc1* may have additional roles in regulating lipid metabolism and may act independently of *SpdS* and *Sms* to carry out these additional metabolic functions. Additional experiments aimed at determining the genetic and biochemical interactions that may occur among Odc1, SpdS and Sms in the fly fat body, including determining the levels of each of the polyamines in fat body cells with decreased *Odc1*, *SpdS* or *Sms*, may provide insight into the roles of these enzymes in regulating lipid homeostasis.

Here, we have shown that *SpdS-RNAi* and *Sms-RNAi* flies have normal head–thorax and abdomen fat body triglyceride levels, despite having increased triglycerides when examining whole flies. This suggests that when *SpdS* and *Sms* levels were decreased in fat body cells, alterations in triglyceride storage occurred in non-fat body cells of the abdomen, such as the ovaries, which store lipids to support reproduction [19]. While abdomen fat body triglyceride storage was normal in *SpdS-RNAi* and *Sms-RNAi* flies, fat body cell number was increased compared to control fat bodies. Interestingly, the fat body cells from these flies were also smaller, as indicated by a decreased protein/DNA ratio, and the combination of more fat body cells that are smaller could explain the normal triglyceride storage phenotype observed in the dissected abdomen fat bodies. It is possible that decreasing *SpdS* or *Sms* may alter fat cell differentiation, proliferation, and/or apoptosis to control fat body cell size and number, because polyamines have been well established to regulate these cellular processes [3,4]. Inhibiting ornithine decarboxylase and spermine synthase in cultured 3T3-L1 preadipocytes blocks differentiation into mature adipocytes [6,7,8]. Moreover, the depletion of spermidine in 3T3-L1 preadipocytes was also shown to inhibit adipogenesis [20]. These findings are inconsistent with the increased fat cell number observed in *SpdS-RNAi* and *Sms-RNAi* flies, suggesting that altering cell proliferation and/or differentiation may not be the mechanism whereby decreased *SpdS* or *Sms* expression may alter fat body cell size or number. It is possible that the loss of *SpdS* or *Sms* in the fat body may decrease apoptosis to result in an increase in the number of fat body cells. Polyamines have been shown to either promote or inhibit apoptosis, depending on the cell type and environmental condition [21,22]. Experiments aimed at measuring cell proliferation and apoptosis in fat bodies with decreased *SpdS* and *Sms* expression and further characterizing the triglyceride storage phenotypes in these flies may help clarify these outstanding questions.

The dysregulation of polyamines has been shown to affect glucose and lipid metabolism and diet-induced obesity in numerous model systems [23]. In fact, increased polyamines in white adipose tissue, liver, or muscle have been linked to resistance to diet-induced obesity [24,25]. Moreover, in mice with diet-induced obesity, increases to either spermidine or spermine were shown to cause weight loss [10]. In the study described here, we have characterized the roles of *SpdS* and *Sms* in regulating the storage of triglycerides in the *Drosophila* fat body, thereby expanding the correlation between the polyamines and lipid metabolism. Due to the high conservation of the polyamine synthesis enzymes in most organisms, from *E. coli* to humans [3], the results described here will hopefully be broadly applicable (especially for those people with Snyder–Robinson Syndrome, a disease which arises from mutations in *spermine synthase*). The results of this study support the role of the polyamine pathway in regulating triglyceride storage and adds to our growing understanding of the genes and pathways important for controlling overall lipid homeostasis. Additionally, these studies highlight polyamines as potential therapeutic targets for metabolic disorders such as obesity and type II diabetes.

## Data Availability

The data presented in this study are available with the article.
